# Technology-Based Methods for Training Counseling Skills in Behavioral Health: a Scoping Review

**DOI:** 10.1007/s41347-022-00252-8

**Published:** 2022-04-05

**Authors:** Molly Magill, Nadine R. Mastroleo, Steve Martino

**Affiliations:** 1 Brown University Center for Alcohol and Addiction Studies, Box G-S121-5, Providence, RI 02912 USA; 2 Department of Psychology, Binghamton University, Binghamton, NY USA; 3 Department of Psychiatry, Yale University, New Haven, CT USA; 4 VA Connecticut Healthcare System, West Haven, CT USA

**Keywords:** Alcohol and other drug treatment, Remote learning, Simulation, Standardized patients, Virtual patient training

## Abstract

In the present review, we consider technology-based methods for training and monitoring counseling skills in behavioral health (i.e., addictions, mental health, and behavioral medicine). We provide an overview of topical foci and design features, as well as review the available research. The Arksey and O’Malley framework for scoping review was used and there were two project phases. First, we reviewed and charted design features and training topics. Second, we reviewed and charted published research evaluating training outcomes. The search process yielded six commercial companies or academic research centers targeting online training of behavioral health counseling skills. These programs could be categorized by an avatar (i.e., computer-generated) or video (i.e., human actor) client interface, as well as by a completely interactive experience (i.e., virtual reality) or an experience with a pre-programmed, branch-logic interaction (i.e., computer simulation). One final company provided monitoring services only, without an explicit training component. The literature in this area is in its nascent stages, with primarily pilot scope and comparatively less progress if contrasted with fields such as general medicine. Online training and monitoring of behavioral health counseling skills is a promising emerging field with positive qualities such as scalability, resource efficiency, and standardization. Future research should emphasize (1) between-group randomized clinical trials, (2) comparisons to standard training practices, and (3) alignment with professional competency standards.

**Supplementary Information:**

The online version contains supplementary material available at 10.1007/s41347-022-00252-8.

## Introduction

Established methods for training clinical providers of evidence-based interventions involve some form of practice-based learning, such as role play with performance feedback. The feedback involves use of competency measures in generalist (e.g., client-centered communication; Tracey et al., 1988) or specific-modality (e.g., motivational interviewing; Moyers et al., 2005, 2016) counseling skills. These core training strategies of practice and feedback have been associated with significant and sustained changes in skill acquisition among both addictions and general psychosocial therapy providers (Herschell et al., 2010; Schwalbe et al., 2014). While effective, this approach requires significant resources, and as such, educators, implementation scientists, and other counselor-training stakeholders have begun to use computer technology to aid training, quality improvement, and performance monitoring in educational and direct care settings (Imel et al., 2017). These technology-based methods have some advantages over traditional face-to-face approaches including scalability, resource efficiency, and standardization (Washburn et al., 2016). There may also be limitations, and foremost could be the acceptability of using computers to train human interactions. This scoping review examines the state of the field in technology-based methods for training and monitoring counseling skills in behavioral health. We define these methods as computer programs that provide interactive experiences to train, assess, and provide feedback on skill-based counseling competencies. We use the term behavioral health to encompass counselor training needs in addictions, mental health, and behavioral medicine.

### Background

In medical education, technology-based training methods are standard practice, but are relatively novel in behavioral health education and direct care settings. In 2011, a meta-analysis of virtual patient simulation training (i.e., computer programs that simulate a clinical scenario where learners adopt the role of provider to obtain a medical history, conduct an exam, and/or make diagnostic decisions) found large effect sizes on knowledge and skill outcomes across 50 studies in medicine, nursing, and dentistry. These effects were in relation to no-intervention comparisons, while effects compared to human simulations were less promising (non-significant to small; Cook et al., 2011). The authors also concluded that programs designed with opportunity for practice and performance feedback were associated with better learning outcomes than products without these design features. More recently, a meta-analysis of 31 studies found moderate post-training effects on cognitive and behavioral skills in contrast to usual health education (Kyaw et al., 2019). Despite the promise of technology-facilitated training in general medicine, we were able to locate only one previous review with applicability to behavioral health (Huttar & BrintzenhofeSzoc, 2020; reviewed below in reference to one virtual reality platform—Second Life).

### Overview and Aims

The scoping review is appropriate when a broad approach is needed. Specifically, the goal is to describe the nature of the literature and clarify key concepts and/or future research questions. A scoping review may also include a more diverse range of sources than a narrowly defined selection of research studies (Munn et al., 2018; Pham et al., 2014). In areas with few randomized controlled trials, which limits researchers’ ability to conduct a systematic review, scoping reviews can describe the nature of the available evidence and provide a foundation for future research (Arksey & O’Malley, 2005). The primary aim of this scoping review is to identify and describe technology-based methods for training behavioral health counseling skills. We review available commercial and non-profit entities that train *generalist* (e.g., empathy; client-centered communication) or *specific-modality* (e.g., motivational interviewing; screening and brief intervention) counseling skills. We additionally consider programs that strictly target performance monitoring, given the role of performance monitoring in facilitating training outcomes. Using Kirkatrick’s (1994) Model for typologizing training outcomes, we also review available studies that have evaluated these programs in relation to changes in trainee *reaction* (i.e., attitudes such as acceptability or self-efficacy) and *learning* (i.e., post-training mastery such as knowledge or skills). As educational and intervention methods become increasingly facilitated by technology, behavioral health providers and behavioral health educators need accessible knowledge on available training options. These options have become particularly valuable within the remote learning context of the COVID 19 pandemic, although this need is likely to continue. We are aware of no prior reviews on this topic and therefore, this work addresses an important gap in the training and quality improvement literature.

## Methods

### Problem Statement and Research Questions

A key challenge in behavioral health provider education is how to effectively train and monitor a range of counseling skills with acceptability, scalability, and cost-effectiveness. Despite progress in fields such as general medicine, technology-based training methods in behavioral health are in their infancy. As such, the guiding research questions for this scoping review are:

1. What technology-based programs are available for training and monitoring counseling skills in behavioral health?
2. What is the nature of the outcome research on these programs in relation to reaction and learning outcomes?

### Search Process and Inclusion Criteria

The review required two phases of source searches, conducted by the first author with expert consultation provided by the authorship team. For the Question One search (program overview), a review of program websites was conducted. Inclusion criteria targeted the following: (1) technology-based methods for counseling skills training and/or performance monitoring; (2) counseling skills included diagnostic interviewing, client-centered communication skills, and other clinical skills that could have applications in behavioral health; (3) behavioral health was used as an umbrella term to encompass addictions, mental health, and other behavioral medicine counseling; (4) the programs were English language, and (5) the programs were available for use in educational and direct care settings. When a website with eligible programs was identified, a 30 to 60-min meeting with a company or organizational representative was conducted to demonstrate the online program and obtain any information that was not readily available via the website. Nine identified companies were excluded because they did not meet inclusion criteria (ActiV; Body Interact; I Human; Medic; Medscape; Shadow Health; STRIVR; Udemy Business; Will Interactive). Specifically, these programs did not target counseling skills applicable to behavioral health contexts, and rather were medical-reasoning focused or targeted human resource and management training in industry.

For the Question Two search (outcome research), we reviewed program websites and identified studies within the “research” or “case study” tabs. We additionally conducted our own independent search for studies in PubMed. Inclusion criteria targeted studies that (1) were peer-reviewed training outcome research, (2) were relevant to behavioral health, (3) reported attitudinal, knowledge, and/or skill outcomes, and (4) were English language publications. The independent search was performed via keyword and by company or research center name (Date range 2000 to October 2021) as well as a reference search of any included studies. The goal was to offer an overview of the available evidence consistent with the general framework for a scoping review. For reviews of a similar nature in medicine, the following references can be considered: Consorti et al., 2012; Cook et al., 2011; Kyaw et al., 2019; Lee et al., 2020. For reviews in behavioral health, we are aware of only one manuscript on a single virtual reality platform—Second Life (Huttar & BrintzenhofeSzoc, 2020).

### Review and Charting Methods

Similar to our search methods, each question required a different review approach. For Question One (program overview), six characteristics were charted and narratively described. First, Industry Status identifies program developers as for-profit or non-profit entities. Second, Topical Focus provides an overview of the targeted skills or topical areas for training. Third, Trainee Type describes the types of trainees that the programs may best target, and this information was derived primarily via a review of characteristic four—Program Examples. Fifth was Program Description, which provides an overview of key training features and underlying technology. Sixth was Costs, where we briefly identify fee structures for purchase (e.g., by students, trainers, or administrators) as well as development (e.g., by educators or researchers). Data collection occurred via a review of websites, program demonstrations, and interviews with a company or center representatives. For Question Two (research review), we took a narrative approach emphasizing a description of trainee population, training topic, study design, and outcomes.

## Results

### Technology-Based Methods for Training and Monitoring Counseling Skills

Our scoping review of technology-based methods for counseling skills training and monitoring in behavioral health yielded commercial companies and research centers spanning health, mental health, addictions, education, defense, and corporate fields. Most entities were unified by their basic technological profile, rather than by the type of client served. Therefore, the same company or center training screening and brief intervention for alcohol use might also be training inclusive corporate practices in industry. What follows is an overview, organized by each company or research center, describing sample programs and program characteristics (see Table 1 for program chart). We categorize these training programs by a computer avatar interface (4 of 6) or video interface (i.e., human actor; 2 of 6), and then discuss one final entity providing monitoring services only.

**Table 1:** Charting technology-based tools for counseling skills training and performance monitoring

	**Industry status**	**Topical focus**	**Trainee type**	**Program types**	**Program description**	**Cost**
***Avatar-interface training and performance monitoring***						
Institute for Creative Technologies	University-based/non-profit	- Defense - Education - Healthcare	- Health and mental health care providers - Military personal - Peer supporters - Students - Teachers	University-based research center. Case study examples include *ELITE Counseling; Motivational Interviewing Learning Environment and Simulation; Motivational Interviewing Novice Demonstration; Stress Resilience in Virtual Environments*	The center offers a wide range of programs for research and direct care purposes. Specific program designs range from immersive virtual reality experiences for therapeutic purposes (e.g., PTSD exposure therapy) to simulated healthcare and leadership scenarios for training purposes. Use options include opportunities for practice with performance feedback	- Costs associated with use in research
Kognito	Private/for profit	- Education - Healthcare - Defense	- Health and mental healthcare providers - Peer supporters - Students - Teachers	26 available training programs. Examples include *At-Risk Mental Health for Students; Behavioral Health, Drug and Alcohol Use; SBI with Adolescents; SBI with Adults*	An avatar-interface, simulation-based program for training skills in healthcare conversations. Key program features include role play conversations, multiple-choice format responses, opportunity for practice, in vivo coaching and performance feedback	- Institutional subscription - Individual license - Developmental agreement
Mursion (TeachLivE)	Private/for-profit university-based/non-profit	- Corporate - Education - Healthcare - Defense	- Corporate managers - Health and mental health care providers - Students - Teachers	Client-company partnership to meet client needs. Website provides case study examples including mental health, cultural competency and inclusion, K-12 education, and corporate leadership	An avatar-interface, virtual reality-based program for practicing essential interpersonal skills and learning how to manage difficult conversations. The program design includes virtual conversations that are fully interactive based primarily on human intelligence	- Managed service - Annual license - Developmental agreement
Second Life	Private/for profit	- Education - Healthcare	- Students - Health and mental healthcare providers	Numerous case study examples are available, including training in clinical interviewing group therapy and group decision- making case management and diversity and inclusion	An open access virtual reality game. Users can join, create an avatar identity and interact in virtual worlds. Teachers in health and mental health have adapted the program for teaching a range of problem- solving skills. Interactions are driven remotely by users, and educational goals would need to be designed by the instructor	- Free with costs for upgraded usage
***Video-interface training and performance monitoring***						
SIMmersion	Private/for profit	- Corporate - Education - Healthcare - Defense	- Health and mental healthcare providers - Law enforcement - Military personal - Students - Teachers	24 available training programs. Examples include *Alcohol Screening and Brief Intervention; Motivational Interviewing; Cognitive Behavioral Therapy: Creating a Change Plan; Cognitive Behavioral Therapy: Functional Analysis*	A video-interface, simulation-based program for training skills in healthcare conversations. Key program features include role play conversations with filmed actors, spoken or multiple-choice format responses, opportunity for practice, in vivo coaching and performance feedback	- Annual license
Theravue	Private/for profit	- Counseling - Psychotherapy	- Students	An industry/expert partnership developed specifically to aid counselor education. The program is designed to address broad counseling competencies and each instructor will assemble content from a selection of over 500 video vignettes	A video-based, *deliberate practice* program for use in counselor and psychotherapy education contexts. Each instructor will “build” a course by selecting from the available video-patient prompts (e.g., *respond to a therapeutic rupture).* Each student will respond to the prompt via a video-recorded role play reaction. Both student and instructor can evaluate the recorded response based on score-rubrics	- Per-student license - Free to instructors
***Performance monitoring***						
Lyssn	Non-profit	- General counseling skills - Motivational interviewing - Cognitive behavioral therapy	- Health and mental health care providers and supervisors - Students	The company offers a service that will facilitate training or supervision of general counseling, motivational interviewing, or cognitive behavioral therapy skills	An online platform for review of session video recordings. Via a monthly, per provider fee, sessions can be recorded, encrypted, and then reviewed with speech to text translation and artificial intelligence analytics	- Monthly service fee

#### Avatar-Interface Training Programs

The first avatar-interface programs are provided by Mursion, whose predecessor is TeachLive from the Center for Research in Education and Simulation Technologies at the University of Central Florida. These programs offer a fully interactive training experience, were originally designed to train elementary and high school educators, but have recently expanded to include some mental health content. In the training, the user is presented with a scenario, a learning goal, and enters a role play with an avatar driven by a human *simulation specialist* (e.g., in the demonstration, the first author interacted with a male client exhibiting manic symptoms and the task was to show patient engagement and rapport building). Because of the human trainer, the technology can be considered an online platform for standardized patient training. Available training topics include health, mental health, education, and corporate leadership experiences, and are typically developed to meet specific goals and objectives identified by the client. Design features include a virtual environment, skill-based competencies to demonstrate, a role play interaction, and immediate performance feedback provided by the simulation specialist.

Other avatar-interface programs provide an interactive training experience not via a human trainer as noted above, but instead via a pre-programmed computer simulation. Specifically, the user selects among verbal inputs that can be qualified by the type of skill (e.g., build rapport, ask question), and the selection cues an algorithm-derived client response (e.g., the avatar remains engaged in the session or withdraws and becomes resistant) in what is termed an *adaptive user experience*. For example, Kognito offers “off-the-shelf” simulation trainings on health, mental health, addictions, and education topics that are all based on the company’s core instructional design and conversational model (Albright et al., 2016). In *Change Talk: Childhood Obesity (open access demonstration: *https://go.kognito.com/changetalk*)*, the user watches a didactic video on a set of motivational interviewing (MI) skills (i.e., open questions, respectful information sharing, using reflections) and then engages in three 10-min simulated patient-provider conversations related to nutritional health. The user can select among text options, while also receiving coaching tips, and/or viewing a patient “thought bubble” about the experience. At the end, the *performance dashboard* provides a standardized score and recommendations for performance enhancement.

Another computer simulation program is the *Motivational Interviewing Learning Environment and Simulation* (see also *Motivational Interviewing Novice Demonstration*), which are both created by the Institute for Creative Technologies (ICT) at the University of Southern California. This training provides students an opportunity to practice MI when treating active military, veterans, and military-impacted families. Performance feedback is provided for 10 skill dimensions (e.g., affirmations, reflective listening, cultivating change talk, rolling with resistance) and can be generated at the individual student and classroom levels. Although not discussed here, the ICT website (https://ict.usc.edu/prototypes/pdf-overviews/) identifies several other training programs targeting communication and leadership skills in a military context.

The final avatar-interface program is Second Life, which is not a training tool per se, but an open access virtual world. In Second Life, users can create their own avatars, explore environments, socialize (i.e., via voice or text chat), and participate in individual and group activities. A modeling tool and scripting language are built into the software, which allows users to build functionality. Due to its open access status and adaptability, numerous learning environments have been constructed in Second Life, including health education for the public, university “builds” with varied teaching objectives, governmental science organizations, and museums (Beard et al., 2009). Potential learning tools include an opportunity for interaction, immersive experiences, role plays, and a set of relevant examples is provided in our outcome research review.

#### Video-Interface Training Programs

SIMmersion is a company offering a video, rather than avatar interface, but produces computer simulation training programs similar to those described for Kognito and Institute for Creative Technologies above. The company addresses a range of training topics, but currently has 18 of 24 available programs specific to behavioral health counseling (e.g., motivational interviewing training, screening and brief intervention training, and suicide prevention). For example, *The Cognitive Behavioral Therapy (CBT) Suite for Substance Abuse* involves three encounters with three different clients, and learning targets include orientation to the cognitive behavioral model, rapport building, and completion of a functional analysis. In all three scenarios, there is didactic content, a patient simulation, coaching, and performance feedback. The client character also has multiple versions and each version varies with respect to motivational status and willingness to engage in behavior change discussions. As a result, multiple practice sessions will yield a different training experience. For performance feedback, client engagement and coach reactions offer feedback in vivo, and an *after-action review* provides standardized scores of CBT adherence and competence.

Theravue has functionality that is quite different from the training programs described thus far. The company website describes Theravue as a *deliberate practice educational platform*. Deliberate practice can be applied to any skill development, but has become of increasing interest in the psychotherapy field as an ongoing approach to improving therapeutic effectiveness (Rousmaniere, 2017; Rousemaniere et al., 2017). With Theravue, the student or practitioner will engage in a focused and ongoing practice, and performance feedback occurs via self- or instructor assessment. Trainees self-select filmed vignettes or complete a series of vignettes designed by the instructor or supervisor. Vignette examples are numerous and include modules (i.e., a selection of video vignettes and corresponding evaluation rubrics) on clinical competencies such as empathy, summaries, open-ended questions, and psychoeducation. A search function allows case selection by age, gender, race or ethnicity, and diagnosis or other presenting clinical conditions. For example, in an *Empathy Vignette*, an adult male with depression describes his daily routine; the trainee can video-record several responses prior to selecting one to submit for review by the instructor or clinical supervisor. For performance feedback, the rubric contains roughly five questions such as: “Does the response describe what it is like to be the client?” Instructors can build courses characterized by a broad skill competency (e.g., developing a therapy relationship) or patient profile (e.g., substance use).

#### Performance Monitoring Programs

The final program does not target training, but rather supervision or other quality control. Given the centrality of therapy session file review in provider training for specific evidence-based modalities, Lyssn may also facilitate training in various educational or direct service contexts. We frame Lyssn as similar to Theravue or Mursion (above), in that the role of the technology is to facilitate the activities of, rather than replace, a human trainer. For technological profile, Lyssn uses speech recognition and machine learning to provide standardized performance feedback on therapy sessions recorded and/or accessed via the program. Assessment dimensions include those highlighted in the Motivational Interviewing Treatment Integrity Scale (Moyers et al., 2005, 2016) as delivered via the *CORE-MI* assessment (e.g., empathy, percent open questions and complex reflections, and reflection to question ratio; Gibson et al., 2017). Lyssn also provides performance metrics for Cognitive Behavioral Therapy (e.g., agenda setting, providing feedback, reviewing homework) based on the Cognitive Therapy Rating Scale (Goldberg et al., 2020). The user uploads or records a session, which ensures encrypted transfer and storage, and an automated transcript and score is produced.

### A Review of Outcome Research

Among the companies, centers, and corresponding programs reviewed, there was significant variability in the extent of peer-reviewed research, types of trainee populations, and evaluation methodology. What follows is a narrative review of outcomes studies, and we follow the general order of the descriptions in the above section (see Supplemental 1 for complete evidence chart).

#### Avatar-Interface Studies

For Mursion (https://www.mursion.com/case-studies/), peer-reviewed research was available in the field of education with one application found in peer counseling. A 2018 review of four studies suggested within-group changes in specific communication and classroom management skills such as giving praise, behavioral prompts, and differential reinforcement of prosocial behaviors, but sample sizes were typically small (i.e., less than 10 participants; Peterson-Ahmad et al., 2018). In a 2021 systematic review, 23 studies were obtained of which 10 were classified as outcome studies. Within these, the majority were within-group, pre-post designs, or quasi-experimental between-group designs. In addition, the majority of studies targeted pre-service trainees (i.e., student teachers) and included assessment of specific teaching skills, classroom management skills, and interviewing skills (Ersozlu et al., 2021). Recently, a quasi-experimental between-group study examined a Mursion peer-coaching simulation among 79 Division 1 NCAA athletes. Each participant received three simulated encounters focused on having difficult conversations about playing time, stress and anxiety, and life-sport balance. In pre-post design, the Mursion participants did not show differential changes on an interpersonal communication competency measure compared to a no-treatment control (Fraley et al., 2020).

Kognito (https://kognito.com/resources?resource_type=Research) programs have an emerging body of pilot evaluation research in the behavioral health context. In an evaluation of the *Screening and Brief Intervention (SBI) Skills Assessment* simulation, a sample of 423 bachelor- and master-level social work students were exposed to an enhanced curriculum in field, generalist, and elective courses, including the use of the simulated training program. Although study results evaluated the entire curriculum enhancement, 61% of students achieved mastery in the targeted engagement and assessment skills (Boyle & Pham, 2019). Another study with nursing and social work students examined the effects of the SBI simulation directly. The sample (*N* = 108) received a 30-min lecture and then completed the *SBI with Adolescents* simulation in a pre-post, within-group design. The study reported increases in self-reported confidence and readiness to engage with adolescents who use substances (Hitchcock et al., 2019), although changes in student skills were not assessed. Similar within-group, self-report results on attitudes (i.e., confidence, readiness) were observed with 144 undergraduate nursing students (Burmester et al., 2019). In a recent trial on *At-Risk for College Students*, 117 undergraduates were randomized to the peer suicide and mental health referral training or to a waitlist control. The study found differential increases in referral preparedness and self-efficacy at post-treatment, and in actual peer referrals at 1-month follow-up (Coleman et al., 2019). Two within-group studies provide further support for this programs’ effect on mental health referral attitudes among both peers and university staff counselors (Rein et al., 2018; Smith-Millman et al., 2020).

A review of the Institute for Creative Technologies’ publication page shows a large body of research spanning 10 years, but these studies pertain to a range of topics such as program development, virtual reality platforms for mental health treatment (e.g., post-traumatic stress disorder), and emerging work evaluating available counselor training programs (https://ict.usc.edu/publications.php). For example, a 2016 pilot study examined the feasibility and effectiveness of a simulated diagnostic assessment among five social work students. Following a brief orientation to the system, participants were told they would complete a series of 30-min diagnostic interviews focused on mental health or substance use concerns. Outcome measurement occurred via review of the first and final virtual patient interview in a pre-post, within-group design. The study found improvements in diagnostic accuracy and interviewing skills and general acceptability despite some difficulties with the speech recognition functionality (Washburn et al., 2016). In another study by the same research team, 22 social work students were randomized to three virtual patient sessions with a virtual or actor patient, or to a no practice control group. The study required a 30-min diagnostic interview on mental health and substance use symptoms, and considered program usability, interview self-efficacy, and diagnostic accuracy as study outcomes. The results showed significant within-group increases in diagnostic accuracy and student self-efficacy in both the virtual and actor patient evaluation conditions relative to control (Washburn et al., 2020). A 2020 study of 122 practicing Veteran’s Administration providers examined the effect of a computer simulation to train motivational interviewing skills (i.e., *Motivational Interviewing Novice Demonstration*) in comparison to a self-study control. The study found effects on three Motivational Interviewing Treatment Integrity indicators (i.e., technical and relational scores, question to reflection ratio) that were maintained up to a 3-month follow-up (Reger et al., 2020).

Educational applications for Second Life have been quite diverse. In fact, a review of research on virtual education found that Second Life applications accounted for 99 of the 167 studies reviewed. Reported learner gains across all studies included changes in self-confidence, communication skills, problem-solving, and empathy (Reisoğlu et al., 2017). A review of 12 qualitative and quantitative studies of Second Life training in nursing education reported acceptance among users, as well as changes in communication and behavioral skills (Irwin & Coutts, 2015). In social work, a systematic review of five evaluation studies sought to describe the nature of virtual education in that field. Training targets included case management (Levine et al., 2013; Wilson et al., 2013), interviewing skills (Tandy et al., 2017), and diversity and inclusion competencies (Lee et al., 2014; Reinsmith-Jones et al., 2015). The review in social work concluded that while the research was typically pilot in nature, there is emerging evidence for acceptability when delivered as an addition to classroom instruction (Huttar & BrintzenhofeSzoc, 2020).

#### Video-Interface Studies

SIMmersion has been evaluated in the fields of law enforcement, defense, education, healthcare, and behavioral health. The latter will be described here. In pilot work, 22 social work students were given access to two cognitive behavioral and one motivational interviewing computer simulation program as part of an enhanced practice course. Usability and acceptability outcomes showed sustained program engagement, self-reported acceptability, and changes in simulation scores in a within-group, pre-post design (Smith et al., 2020). Another pilot study of practicing healthcare providers (*N* = 20) showed similar acceptability, engagement, and changes in knowledge scores for suicide assessment (O’Brien et al., 2019a, b). Of the remaining studies on SIMmersion programs, between-group randomized designs have been used. For example, in a trial of 102 healthcare providers, participants were assigned to at least 10 practice role plays with *Alcohol Screening and Brief Intervention (SBI)* or to a no training control group. The primary outcomes were clinical skill changes assessed via standardized patient role play at baseline and then at a 6-month follow-up. The study found that relative to control, training group providers showed increases in proficiency along screening, assessment, and referral skill dimensions (Fleming et al., 2009). Another study comparing *Cognitive Behavioral Therapy: Introducing CBT* to a therapy manual control condition with 65 social work or psychology students utilized a similar standardized patient, pre-post design. The study found differential increases in extensiveness and skillfulness ratings for skills in agenda setting, explaining CBT concepts, and checking understanding of CBT concepts (Mastroleo et al., 2020). However, when *Alcohol SBI* was added to an in-person training with 308 social work and nursing students, additive effects on self-reported knowledge and skill measures were not observed (O’Brien et al., 2019a, b). The remaining video-interface company (i.e., Theravue) did not have peer-reviewed evaluation data that we could locate via website case studies, direct interview methods, or our independent search.

#### Performance Monitoring Studies

In the performance monitoring company Lyssn, research to date has been focused on development, proof of concept, and acceptability. The company website publication page (https://www.lyssn.io/research) shows studies evaluating performance monitoring accuracy in relation to human raters of therapy sessions. For example, the natural language processing model used to detect specific clinical skills (i.e., open- and closed-ended questions, affirmations, and giving information) from text data has shown at least “good” interrater agreement with trained human raters (Tanana et al., 2016). In an evaluation of usability and acceptability of the automated CORE-MI assessment, 21 providers submitted 10-min standardized patient practice sessions for review. A high percentage of users (i.e., greater than 80%) reported ease of use and intention to adopt the technology in their own practice (Imel et al., 2019). Finally, members of the same research team have begun to test the technology as an automated training tool—ClientBot. In an initial test with 151 non-clinicians, participants were randomized to an automated, text-interface counseling skills training program or to a no-intervention control. The program targeted the use of open-ended questions and reflections with a text-based *conversational agent*. The study found increases in the use of these skills during the active coaching phase relative to control (Tanana et al., 2019).

## Discussion

In the present scoping review, we sought to provide an overview of technology-based programs for counseling skills training and performance monitoring in behavioral health. The goal was not to select one program for endorsement, but rather to offer practitioners, educators, trainers, and implementation scientists a broad view to guide their own decision-making. While working on the project, the unprecedented COVID-19 global pandemic occurred, and as a result, we believe the content of this review may be particularly pertinent to educators and trainers currently in the field. However, there may be advantages that will outlast any immediate need to select an online over an in-person format for behavioral health counseling skills training. These advantages include scalability (Imel et al., 2017), resource efficiency (Washburn et al., 2018), standardization (Alanazi et al., 2017), and immediacy of individualized performance feedback (Tanana et al., 2019). These programs are currently available and they have the capacity to train general counseling skills such as diagnostic interviewing, empathic communication, suicide prevention, and group collaboration or facilitation, as well as specific-modality clinical skills such as screening and brief intervention, motivational interviewing, and cognitive behavioral therapy. With that said, evaluation of these programs is in its infancy, and progress is needed to demonstrate non-inferiority to in-person training methods as well as alignment with professional accreditation standards or other evidence-based practice guidelines (see Supplement 2 for examples). What follows is a discussion of some final considerations for those who may be planning their technology-based training needs.

### Presence Versus Scalability?

This scoping review revealed some program characteristics that could represent a trade-off decision, due to limitations in the available technology. These relate to the use of avatars versus filmed actors as the client interface and the use of a pre-programmed, simulated encounter versus a remote, human simulator. Both considerations relate to *presence*, which refers to the capacity of a virtual reality or computer simulation program to mimic the experience and thus emotional and cognitive arousal of a human interaction (Lee, 2004). The more authentic the experience, the stronger the neural network of skill development (Andresen et al., 2000). In the current review, the programs that could be fully interactive were driven by human simulators, which provides an immediate trade-off with scale. Quite simply, any educational program requiring human input will require greater resources than a program driven entirely by a computer. The advantage, however, is presence, unpredictability, and adaptability. The educator or trainer must, therefore, prioritize programs that meet their immediate training needs. For example, with novice learners, the off-the-shelf programs made by Kognito, SIMmersion, or the Center for Creative Technologies are likely sufficient. In contrast, those with more advanced or specialized training needs may wish to enter into developmental contracts with companies such as Mursion to create a virtual reality experience highly specific to their training context. As time progresses, however, it is likely that artificial intelligence technology will evolve toward increasingly authentic conversational experiences between humans and computers (Hirschberg & Manning, 2015), and trade-offs of presence and scale could become a problem of decreasing relevance.

### Training, Practice, or Performance Monitoring of Which Skills?

Another consideration is the desired training function of the program within the educational context. Are the trainees novice students who need to be taught the basic skills in client-provider communication? In such cases, the programs from the Center for Creative Technologies and Kognito may be a good fit. If both training and practice are desired, the range of personalities within a single SIMmersion client ensures more opportunity for practice than programs with a more static client profile. If deliberate practice among novice or experienced providers is needed, then Theravue offers an opportunity for recording and self-assessment based on standardized skill rubrics across a large number of commonly encountered clinical scenarios. Providers may also be practicing in the field and only performance monitoring is needed (e.g., Lyssn). Additionally, there is the question of what skills must be taught, and while our review shows some breadth of content, it weighs toward general communications skills (e.g., question and active listening skills), which intersect with the skills of motivational interviewing. As a result, training in other evidence-based, specific-modality skills is an important development need for the future. Finally, learning behavioral skills with either live or technology-based training requires opportunities for repeated practice, feedback, and coaching, which most of the programs provide. However, understanding factors, whether individual or institutional, that maximize users’ sustained engagement and adherence should be the focus of future investigations.

### Limitations, Implications, and Conclusions

Limitations of this study must be considered, and as such we characterize our work as a preliminary scoping review of an emerging field. The research review is a representative overview with an emphasis on studies with potential application to behavioral health counseling competencies, and not an exhaustive systematic review of all available research on these programs. Our findings illustrate this is a nascent field (e.g., all studies have been published in 2015 or later) with designs characterized by small samples and within-group, non-experimental designs. The trainee populations also varied and were most often students in college or university settings. As a result, proof-of-concept research for a specific program with a specific trainee population might not be available. This is both a limitation and a research implication. It is the case that more between-group, randomized trials are needed to demonstrate non-inferiority to usual training methods. In medicine, this empirical question has been examined and is still open for debate (Cook et al., 2011; Kyaw et al., 2019). While we offered breadth in the review, there were several programs in medicine, industry, and defense that were excluded based on training content outside of the current scope, as were training programs that were strictly knowledge (e.g., webinars, training portals for evidence-based therapies), rather than skill-based. The interested practitioner, educator, trainer, or implementation scientist may want to consider these programs prior to making any selections. Finally, and returning to Kirkpatrick’s Model for typologizing training outcomes, the data reviewed here show research on *reaction* (i.e., attitudes) and *learning* (i.e., knowledge and/or skill), but not *behavior* (i.e., application in practice) and *results* (i.e., organizational outcomes). Therefore, these later outcome metrics specific to changes to trainee direct practice and program outcomes are important implications for further study.

Training and monitoring of specific counseling skills are central to quality care in behavioral health. In the present scoping review, we found progress in the online environment to be promising, but with gaps in development and evaluation testing. These programs train both general and modality-specific counseling skills and vary in opportunity for new learning, practice, and/or performance monitoring. They also differ by an avatar- or video-based interface and by a degree of authenticity or presence. When selecting a program for use in various contexts, the extent that the program has been validated in that specific setting will be a key consideration. As such, future research should emphasize (1) between-group randomized clinical trials, (2) comparisons to standard training practices, and (3) alignment with professional competency standards.

## Supplementary Information

Below is the link to the electronic supplementary material.

Supplementary file1 (DOCX 34 KB)

Supplementary file2 (DOCX 17 KB)
