# Supplementary material for: Technology-Based Methods for Training Counseling Skills in Behavioral Health: a Scoping Review
Source: J Technol Behav Sci. 2022 Apr 5;7(3):325–36. doi: 10.1007/s41347-022-00252-8 (PMC8983031; doi:10.1007/s41347-022-00252-8)
Supplement: Supplementary file 1 — Supplementary file1 (DOCX 34 KB) [file 41347_2022_252_MOESM1_ESM.docx]

|  | **Citation** | **Trainee Population (*N)*** | **Training Topic** | **Study Design** | **Outcomes** |
| --- | --- | --- | --- | --- | --- |
| ***Avatar-interface training and performance monitoring*** | | | | | |
| Institute for Creative  Technologies^1^  Kognito^2^  Lyssn ^3^  Mursion^4^  (TeachLivE)  Second Life^5^  SIMmersion^6^  Theravue^7^ | Washburn et al., 2016  Washburn et al., 2017  Reger et al., 2020  Boyle et al., 2019  Hitchcock et al., 2019  Burmester et al., 2019  Coleman et al., 2019  Smith-Millman et al., 2020  Rein et al. 2018  Tanana et al., 2019  Imel et al., 2019  Tanana et al., 2019  Fraley et al., 2016  Peterson-Ahmad et al., 2018 *(review)*  Ersozslu et al., 2021  *(review)*  Reisoğlu et al., 2016  *(review)*  Irwin & Coutts, 2015  *(review)*  Huttar & BrintzenhofeSzoc, 2020  *(review)*  Smith et al., 2020  O’Brien et al., 2019  Fleming et al., 2009  Mastroleo et al., 2020  O’Brien et al., 2020  --- | social work students (*N* = 5)  social work students (*N* = 22)  VA providers (*N* = 120)  social work students (*N* = 423)  social work and nursing students (*N* = 108)  nursing students (*N =* 144)  college student peer counselors (*N* = 117)  college student peer counselors and staff (*N* = 210)  college student peer counselors and staff (*N =* 2727)  motivational interviewing providers (*N* = 341)  motivational interviewing providers (*N* = 21)  novice providers (*N =* 151)  Student Athletes (*N =* 79)  teaching students, teachers (*K =* 4)  teaching students, teachers (*K =* 10)  college students (*K =* 99)  nursing students, including mental health nurses (*K =* 14)  social work students (*K =* 5)  social work students (*N =* 22)  healthcare providers (*N* = 308)  healthcare providers (*N* = 102)  social work and psychology students (*N* = 65)  social work and nursing students (*N =* 308)  --- | addictions/mental health interviewing  addictions/mental health interviewing  Motivational Interviewing Novice  Demonstration, addictions  Screening Brief Intervention (SBI), addictions  SBI with Adolescents, addictions  SBI with Adolescents, addictions  At Risk College Students, mental health  At Risk College Students, mental health  At Risk College Students, mental health  automated versus human rated sessions  CORE MI Assessment  ClientBot  peer simulation, mental health  classroom simulation, education  classroom simulation, education  a range of reasoning and task-based training environments^*^  a range of reasoning and task-based training environments^*^  a range of task-based and experiential learning environments^*^  cognitive behavioral therapy, motivational interviewing, addictions  suicide assessment, mental health  Screening Brief Intervention (SBI), addictions  Cognitive Behavioral Therapy -Introducing CBT, addictions  Screening Brief Intervention (SBI), addictions  --- | within group  between group, randomized  between group, randomized  within group, part of an enhanced course  within group  within group  between group, randomized  within group  within group  within group  within group  between group, randomized  between group  within group  within group, quasi-experimental between group  case study, within group, quasi-experimental  within group  within group  within group  within group  between group, randomized  between group, randomized  between group, randomized  --- | acceptability; diagnostic accuracy; interviewing skill  acceptability; diagnostic accuracy; interviewing self-efficacy  Motivational Interviewing Treatment Integrity scores  engagement and assessment skills  competence, confidence, readiness to engage with adolescents who use substances  competence, confidence, readiness to deliver SBI  referral preparedness, self-efficacy, number of referrals  referral preparedness, self-efficacy  referral preparedness, self-efficacy  good agreement in motivational interviewing skills; poor agreement on client change talk  acceptability, usability, intention to adopt  questions and reflections  communication skills  classroom management skills, including praise, prompts, and differential reinforcement  classroom management, peer coaching, interviewing skills  a broad range of cognitive and behavioral learner gains reported  acceptability, interviewing skills, conflict resolution, and behavioral tasks related to medical interventions  acceptability, case management, interviewing and assessment skills, self-awareness around bias and discrimination  program engagement, acceptability, simulation scores  program engagement, acceptability, simulation scores  SBI skills  CBT skills  no additional effect over in-person training on SBI knowledge and skill  --- |

*Notes. N =* number of participants, *K =* number of studies.

^1^ Search by PubMed and <https://ict.usc.edu/publications.php?all=1&page=17&bib=ICT.bib> with keywords addiction, mental health, behavioral health. 146 articles screened (inclusion: 1) peer reviewed training outcome study, 2) relevant to behavioral health, 3) reported outcomes specific to acceptability and/or effectiveness, and 4) were English language publications).

^2^ Search by PubMed and <https://kognito.com/resources?tresource_type=Research> with keywords substance use, mental health, suicide prevention. 62 articles screened (inclusion: 1) peer reviewed training outcome study, 2) relevant to behavioral health, 3) reported outcomes specific to acceptability and/or effectiveness, and 4) were English language publications).

^3^ Search by PubMed and https://www.lyssn.io/the-science/#academic-papers. 27 articles screened (inclusion: 1) peer reviewed training outcome study, 2) relevant to behavioral health, 3) reported outcomes specific to acceptability and/or effectiveness, and 4) were English language publications).

^4^ Search by PubMed and <https://www.mursion.com/case-studies/> with keywords addiction, mental health, behavioral health. 116 articles screened (inclusion: 1) peer reviewed training outcome study or review, 2) allowed teaching research due to absence of behavioral health studies, 3) reported outcomes specific to acceptability and/or effectiveness, and 4) were English language publications).

^5^ Search by PubMed and https://secondlife.com/. 41 articles screened (inclusion: 1) peer reviewed training outcome study or review, 2) relevant to behavioral health, 3) reported outcomes specific to acceptability and/or effectiveness, and 4) were English language publications). ^*^ These environments typically involved an assigned task where specific reasoning or behavioral skills could be demonstrated. Experiential learning environments included a virtual Holocaust museum and inner city setting.

^6^ Search by PubMed and <https://www.simmersion.com/publications> with keyword healthcare. 44 articles screened (inclusion: 1) peer reviewed training outcome study, 2) relevant to behavioral health, 3) reported outcomes specific to acceptability and/or effectiveness, and 4) were English language publications).

^7^ Search by PubMed and https://www.theravue.com/. No peer reviewed publications found.
