# Supplementary material for: Technology-Based Methods for Training Counseling Skills in Behavioral Health: a Scoping Review
Source: J Technol Behav Sci. 2022 Apr 5;7(3):325–36. doi: 10.1007/s41347-022-00252-8 (PMC8983031; doi:10.1007/s41347-022-00252-8)
Supplement: Supplementary file 2 — Supplementary file2 (DOCX 17 KB) [file 41347_2022_252_MOESM2_ESM.docx]

Supplement 2. Accreditation Standard and Evidence-Based Guidelines relevant to the Programs Reviewed

|  | **Social Work (CSWE)^1^** | **American Psychological Association (APA)^2^** | **Counseling and Related Educational Programs (CACREP)^3^** |
| --- | --- | --- | --- |
| Institute for Creative  Technologies  Kognito  Lyssn  Mursion  Second Life  SIMmersion  Theravue | **Competency 6:** Engage with Individuals, Families, Groups, Organizations, and Communities; **Competency 7:** Assess Individuals, Families, Groups, Organizations, and Communities; **Competency 8:** Intervene with Individuals, Families, Groups, Organizations, and Communities; **Competency 9:** Evaluate Practice with Individuals, Families, Groups, Organizations, and Communities  **Competency 6:** Engage with Individuals*,* Families, Groups, Organizations, and Communities; **Competency 7:** Assess Individuals, Families, Groups, Organizations, and Communities; **Competency 8:** Intervene with Individuals, Families, Groups, Organizations, and Communities; **Competency 9:** Evaluate Practice with Individuals, Families, Groups, Organizations, and Communities  **Competency 6:** Engage with *Individuals,* Families, Groups, Organizations, and Communities; **Competency 8:** Intervene with *Individuals,* Families, Groups, Organizations, and Communities; **Competency 9:** Evaluate Practice with *Individuals,* Families, Groups, Organizations, and Communities  **Competency 6:** Engage with *Individuals,* Families, Groups, Organizations, and Communities; **Competency 9:** Evaluate Practice with *Individuals,* Families, Groups, Organizations, and Communities  **Competency 2:** Engage Diversity and Difference in Practice  **Competency 6:** Engage with Individuals*,* Families, Groups, Organizations, and Communities; **Competency 7:** Assess Individuals, Families, Groups, Organizations, and Communities; **Competency 8:** Intervene with Individuals, Families, Groups, Organizations, and Communities  **Competency 6:** Engage with Individuals*,* Families, Groups, Organizations, and Communities; **Competency 7:** Assess Individuals, Families, Groups, Organizations, and Communities; **Competency 8:** Intervene with Individuals, Families, Groups, Organizations, and Communities  **Competency 2:** Engage Diversity and Difference in Practice;  **Competency 6:** Engage with Individuals*,* Families, Groups, Organizations, and Communities; **Competency 8:** Intervene with Individuals, Families, Groups, Organizations, and Communities | **Competency 1:** Evidence-based Intervention; **Competency 2:** Evidence-based Assessment; **Competency 7:** Communication and Interpersonal Skills; **Competency 10:** Reflective Practice  **Competency 1:** Evidence-based Intervention; **Competency 2:** Evidence-based Assessment; **Competency 7:** Communication and Interpersonal Skills; **Competency 10:** Reflective Practice  **Competency 1:** Evidence-based Intervention; **Competency 7:** Communication and Interpersonal Skills; **Competency 10:** Reflective Practice  **Competency 7:** Communication  and Interpersonal Skills; **Competency 2:** Evidence-based Assessment; **Competency 10:** Reflective Practice  **Competency 4:** Individual and Cultural Diversity; **Competency 7:** Communication and Interpersonal Skills  **Competency 1:** Evidence-based Intervention; **Competency 2:** Evidence-based Assessment; **Competency 7:** Communication and Interpersonal Skills; **Competency 10:** Reflective Practice  **Competency 1:** Evidence-based Intervention; **Competency 4:** Individual and Cultural Diversity; **Competency 7:** Communication and Interpersonal Skills; **Competency 10:** Reflective Practice | **Section 5a Addiction Counseling:** 3 (practice dimensions); **Section 5c Mental Health Counseling:** 3 (practice dimensions); **Section 5e College Counseling and Student Affairs:** 3 (practice dimensions); **Section 5g School Counseling:** 3 (practice dimensions)  **Section 5a Addiction Counseling:** 3 (practice dimensions); **Section 5c Mental Health Counseling:** 3 (practice dimensions); **Section 5e College Counseling and Student Affairs:** 3 (practice dimensions); **Section 5g School Counseling:** 3 (practice dimensions)  **Section 5a Addiction Counseling:** 3 (practice dimensions)  **Section 5g School Counseling:** 3 (practice dimensions)  **Section 5a Addiction Counseling:** 3 (practice dimensions); **Section 5b Career Counseling:** 3 (practice dimensions); **Section 5c Mental Health Counseling:** 3 (practice dimensions); **Section 5e College Counseling and Student Affairs:** 3 (practice dimensions); **Section 5f Rehabilitation Counseling;** 3 (Practice dimensions)**; Section 5g School Counseling:** 3 (practice dimensions)  **Section 5a Addiction Counseling:** 3 (practice dimensions); **Section 5b Career Counseling:** 3 (practice dimensions); **Section 5c Mental Health Counseling:** 3 (practice dimensions) |

*Notes:* Competency designations are based on the availability of programs that are applicable to one or more of the noted competency areas. Evaluation data may not be available for a given competency area and thus program selectors should consider availability of outcome data when selecting a program.

^1^Council on Social Work Education. (2015). *Educational Policy and Accreditation Standards for Baccalaureate and Master’s of Social Work Programs.* CSWE

^2^American Psychological Association. (2015*). Standards of Accreditation for Health Service Psychology and Accreditation Operating Procedures.* APA

^3^Association for Counselor Education and Supervision. (2016). Standards for Accreditation of Counseling and Related Educational Programs. CACREP
